# Role of Yeast Mannoproteins in the Interaction between Salivary Proteins and Flavan-3-ols in a Cell-Based Model of the Oral Epithelium

**DOI:** 10.1021/acs.jafc.1c08339

**Published:** 2022-05-31

**Authors:** A. M. Ramos-Pineda, E. Manjón, R. I. R. Macías, I. García-Estévez, M. T. Escribano-Bailón

**Affiliations:** † Grupo de Investigación en Polifenoles (GIP), Departamento de Química Analítica, Nutrición y Bromatología, Facultad de Farmacia, Universidad de Salamanca, Salamanca, E37007, España; ‡ Natac Biotech S.L., C/Electrónica 7, E28923 Alcorcón, Spain; § Experimental Hepatology and Drug Targeting (HEVEPHARM) Group, Institute of Biomedical Research of Salamanca (IBSAL), CIBERehd, Universidad de Salamanca, Salamanca, E37007, Spain

**Keywords:** astringency, oral cells, mannoproteins, salivary proteins, flavan-3-ols

## Abstract

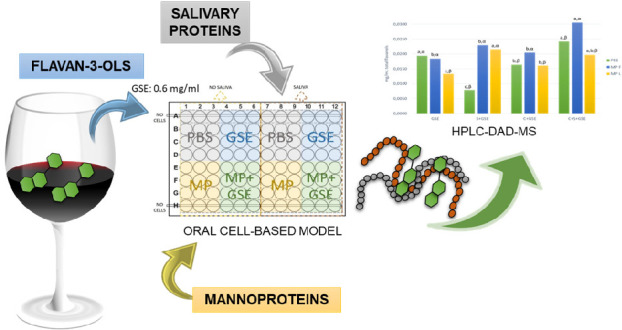

Astringency is a highly complex sensation which involves multiple mechanisms occurring simultaneously, such as the interaction between flavan-3-ols and salivary proteins (SP). Moreover, astringency development can be affected by the presence of polysaccharides such as mannoproteins (MP). The aim of this work was to evaluate the molecular mechanisms whereby MP could modulate the astringency elicited by tannins, using a cell-based model of the oral epithelium (TR146 cells), and the effect of salivary proteins on these interactions. The binding of flavan-3-ols to oral cells was evaluated by DMACA assay, while the content of unbound flavan-3-ols after the interactions was assessed by means of HPLC-DAD-MS. Results obtained confirm the existence of cell–tannin interactions, that can be partially inhibited by the presence of SP and/or MP. The most significant decrease was obtained in the system containing MPF (38.16%). Both mannoproteins assayed seem to have modulating effect on flavan-3-ol–SP interactions, acting by two different mechanisms: MPF would lead to the formation of SP/MPF/flavan-3-ols ternary soluble aggregates, while MPL seems to prevent flavan-3-ol–saliva interaction by a competitive mechanism, i.e., MPL would reduce cell–tannin interactions, similar to SP. This study suggests that mannoproteins with different compositional characteristics could exhibit preferential interaction with distinct flavan-3-ol families.

## Introduction

Tannins are a group of polyphenols with great structural diversity and a wide range of molecular masses that have been classically divided into condensed tannins (proanthocyanidins) and hydrolyzable tannins, which include gallotannins and ellagitannins. Proanthocyanidins are the main tannins found in grapes, being polymers of flavan-3-ol units, that is, (epi)catechins and (epi)gallocatechins. These compounds are mainly present in the grape skin and seeds^1^ and are extracted to the wine during the winemaking process.^2^ Furthermore, the addition of tannins to red wine through commercially available oenological products has widespread acceptance in the wine industry, as they have been reported to make systematic and reliable improvements to the quality of wines.^3^

In particular, the term tannin refers to the property of these compounds to form stable complexes with proteins and other macromolecules, leading to precipitation. This ability seems to be related to the sensation of dryness in the mouth caused by wine and other tannin-rich foods, called astringency, which can be defined as the set of sensations that produce drying, roughing, and puckering of the mouth epithelia.^4^ Thus, astringency can be interpreted as an unpleasant sensation in some foods. However, it is a fundamental attribute in the field of oenology, being even considered a quality parameter of red wines and a major contributor to its consumer acceptance.^5^

Although many researchers have extensively studied the phenomenon of astringency over the years, it is a complex perceptual phenomenon which could involve several sensations that are perceived simultaneously. Indeed, we are still far from elucidating the detailed mechanisms whereby astringency develops.^6^ It is generally considered that astringency is related to polyphenol–protein interactions, because some polyphenols are able to bind salivary proteins, leading to insoluble protein–tannin precipitates in the mouth, causing a loss of lubrication and increasing friction in the oral cavity.^7^

Salivary proteins (SP) have been classified into different groups attending to their structure and characteristics, namely α-amylases, mucins, carbonic anhydrases, statherins, P-B peptide, histatins, cystatins, and proline-rich proteins (PRPs).^8,9^ The PRP family, characterized by a high content of proline, is divided, in turn, into acidic (aPRPs), basic (bPRPs), and glycosylated (gPRPs) proteins.^8,9^ As most of the PRPs are able to precipitate tannins, many of the works published related to astringency have been focused on this protein family, although further studies have revealed that there are other salivary protein families with this ability.^10,11^

Beyond the traditional focus, other approaches have suggested that astringency could involve multiple mechanisms, including the alteration of the mucosal pellicle,^12^ the activation of specific taste receptors,^13^ or even direct interactions between tannins and oral mucosa lipids.^14^ Supporting this last theory, Reis and co-workers investigated polyphenol–lipid interactions in model membranes trying to mimic mouth regions, reporting that lipid microenvironments play a role in oral sensory perception.^14^ Additionally, other studies have suggested that the main role of PRPs is not to precipitate tannins, but they could play a protective role preventing astringent compounds from interacting directly with the oral mucosa.^12,15^ Thus, all the research published over the years has demonstrated that astringency is a highly complex sensation, highlighting the importance of considering more than one mechanism when studying astringency.

To gain a better understanding of this intriguing process, recent studies have focused on developing more realistic cell-based models including the major constituents of the oral cavity that possibly participate in the astringency perception.^12,16−19^ Payne and co-workers led the way, demonstrating that flavan-3-ols present in wine bind to oral epithelial cells in vitro.^16^ Other authors continued studying the role of the oral epithelium through cell culture assays while also considering the effect of salivary proteins on these interactions.^12,17,19^

As aforementioned, although astringency is a quality parameter of red wines, it can be considered nonpleasant when perceived in high intensity. For this reason, the wine industry requires the development of different strategies to modulate harsh astringency. The addition of polysaccharides during winemaking and aging is a practice addressed to correct excessive astringency. There are two main mechanisms proposed to explain the reduction of astringency due to the addition of polysaccharides: (i) the formation of protein/tannin/polysaccharide ternary soluble aggregates^20,21^ and (ii) the preferential interaction between the tannin and the polysaccharide, which competes with the protein and, therefore, inhibits the formation of protein–tannin aggregates.^22^

Mannoproteins represent ca. 35% of total wine polysaccharides.^23^ These polysaccharides are glycoproteins released from the yeast cell wall during alcoholic fermentation and autolysis,^24^ and their influence on sensory quality of wines has been widely reported.^25,26^ Furthermore, a very recent study carried out by Manjón and co-workers^27^ demonstrated, for the first time, a relationship between the compositional characteristics of mannoproteins and their differences in the mechanisms of action toward astringency modulation.

Thus, the main objective of this work was to study, for the first time, the effect of the presence of different mannoproteins, which show structural differences, on the interaction between flavan-3-ols and oral epithelial cells in the presence and absence of salivary proteins. For this purpose, two mannoproteins with different protein percentages (low protein content, MPF, and high protein content, MPL)^27^ have been selected to evaluate the molecular mechanisms whereby they could modulate the astringency elicited by tannins, using a cell-based model of the oral epithelium which also includes the effect of salivary proteins on these interactions.

## Materials and Methods

### Reagents

All reagents used were of analytical grade, and all solvents were HPLC grade. The yeast mannoproteins employed (MPF and MPL, purity >90%) were kindly supplied by Laffort España S.A. (Errenteria, Spain) and LALLEMAND (Fredericia, Denmark). These mannoproteins were obtained from *Saccharomyces cerevisiae* cell walls, and they were described by suppliers to be used in wines for astringency modulation.

Ultrapure water was obtained from a Milli-Q Gradient water purification system (Millipore, Billerica, MA). Dulbecco’s modified Eagle’s medium (DMEM), fetal bovine serum (FBS), and penicillin–streptomycin (P/S) used in cell culture were procured from Gibco by Life Technologies (Thermo Fisher Scientific, Waltham, MA). Phosphate-buffered saline (PBS) 10x was obtained from Sigma-Aldrich (St. Louis, MO). DMACA (4-(dimethylamino)cinnamaldehyde, purity >98%) was purchased from Sigma-Aldrich (Gillingham, UK).

### Grape Seed Tannins

Seeds were separated by hand from the skins and pulp of *Vitis vinifera* L. cv. Tempranillo grapes that were harvested at maturity. Grape seeds were lyophilized and ground to obtain a homogeneous powder. The resulting powder was extracted three times with ethanol/water (75:25 *v*/*v*) for 15 min in an ultrasonic bath. A C18 solid-phase extraction cartridge was used to purify the obtained extract, by eluting with 20% of ethanol, which resulted in a representative mixture of monomeric and oligomeric wine procyanidins.^28,29^ The supernatant of the resulting solution was evaporated to remove the organic solvents and then frozen and freeze-dried. The resulting grape seed extract (GSE) powder was stored at 4 °C until analysis. The content of monomeric and oligomeric flavan-3-ols was determined by HPLC-DAD-MS by using the method described by García-Estévez and co-workers.^30^ The composition of the GSE is shown in Table SI-1 of the Supporting Information (purity >95%). The mDP of the total flavan-3-ols of GSE was 2.05. GSE is composed mainly of monomers and dimers, with a percentage of 25.02% and 46.86%, respectively. The content of galloyl derivatives was 8.17%, while the nongalloylated flavan-3-ols represented 91.82%.

### Saliva Collection and Treatment

Unstimulated whole mouth saliva was collected from seven healthy individuals (27–50 years) who had no history of disorders in oral perception and were not taking any medication. Saliva samples were taken between 10 and 12 am at least 1 h after consuming any food. Samples were collected by expectorating saliva into an ice-cooled tube. All the samples were pooled and centrifuged at 20 700*g* for 10 min at 4 °C to remove any insoluble material. The resulting supernatant was aliquoted and immediately frozen at −80 °C, which is referred to as whole saliva (WS).^31,32^

### Cell Culture

Human oral squamous carcinoma TR146 cell line (ECACC, Porton Down, U.K.) was used in this study as an in vitro model of human buccal epithelium.^33^ TR146 cells were grown in T75 flasks containing Dulbecco’s modified Eagle’s medium (DMEM)/F12 (1:1, *v*/*v*) supplemented with 15% fetal bovine serum (FBS) and 1% penicillin–streptomycin (P/S). The cells were maintained at 37 °C in a humidified atmosphere with 5% CO_2_. When the cells reached 70–80% confluent, the spent medium was discarded, and the monolayer was rinsed with PBS incubated at 37 °C for 5 min with trypsin–EDTA solution to detach the adherent cells. Culture medium was immediately added to the flask and gently mixed to recover the cells. The suspension was then centrifuged at 210*g* for 5 min. The supernatant was carefully aspirated, and the pellet was gently resuspended in culture medium. The cells were diluted with the appropriate volume of culture medium and plated in 96-well plates at a density of 1.5 × 10^4^ cells/well, if grown during 24 h, or 1 × 10^4^ cells/well, if grown during 48 h, before use in an assay.

### Interaction Assays between Flavan-3-ols, Mannoproteins, and Oral Epithelial Cells in the Presence/Absence of Salivary Proteins

TR146 cells were cultured into 96-well flat plates and grown to confluence before use in an assay. The cell monolayers were washed twice with PBS to remove residual culture medium. Stock solutions of GSE and MP were prepared in PBS. At the beginning of the interaction assays, GSE+MP mixture (2:1 *v*/*v*) was incubated at room temperature during 15 min, before adding to the cells. WS (columns 7–12 in Figure S1) or PBS (columns 1–6 in Figure S1) was added at 30 μL/well followed immediately by addition of 15 μL of the interaction mixture (PBS, GSE+PBS, MP+PBS, or GSE+MP, in each case; see Figure S1) (final concentration GSE: 0.6 mg/mL, MP: 2 mg/mL) to a final volume of 45 μL and incubated with the cell monolayer for 15 min at 37 °C. Each assay was repeated in triplicate by using three different wells. After incubation, the supernatant of the cells was removed and centrifuged in Eppendorf tubes for 5 min at 13 709*g*. The pellet was discarded, and the flavan-3-ol composition of the resulting supernatant was analyzed by means of HPLC-DAD-MS. Control solutions of each condition without oral cells were also tested in triplicate (lines A and H in Figure S1).

### DMACA Bioassay

DMACA assay was performed because this compound reacts selectively with catechins and procyanidins to form a blue-green product; thus, the amount of flavan-3-ols that remains bound to the epithelial cells can be quantified.^34,35^ A 0.1% DMACA solution was prepared in acidified methanol (0.75 M H_2_SO_4_) and added to the 96-well plates after the interaction assays. Cells were incubated with 30 μL/well of the reagent for 20 min at room temperature. Finally, the absorbance at 640 nm of each well was determined in a microtiter plate reader.

### Flavan-3-ol Quantification by HPLC-DAD-MS

The content of monomeric and oligomeric flavan-3-ols that remains in the supernatant after the interaction assays was determined by HPLC-DAD-MS analysis after filtering through 0.45 μm pore filters, following the method described by García-Estévez and co-workers.^30^ An Agilent 1100 series HPLC system (Agilent Technologies, Waldbronn, Germany) was employed for the HPLC-DAD analyses, by using an Agilent Poroshell 120 EC-C18 column (150 × 4.6 mm, i.d. 2.7 μm) thermostated at 25 °C as stationary phase. A 0.1% (*v*/*v*) formic acid aqueous solution (solvent A) and HPLC grade acetonitrile (solvent B) were used as mobile phases. A flow rate of 0.5 mL/min and the following gradient were used: from 100 to 90% A for 3 min, from 90 to 85.5% A for 34 min, from 85.5 to 80% A for 3 min, from 80 to 65% A for 15 min, from 65 to 40% A for 5 min, and a final isocratic gradient of 40% A for 3 min. Detection was carried out at 280 nm as the preferred wavelength, and spectra were recorded from 220 to 600 nm. A 3200 Qtrap (Applied Biosystems, Darmstadt, Germany), equipped with an ESI source and a triple-quadrupole linear ion trap mass analyzer controlled by Analyst 5.1 software, was used for MS detection. Zero grade air served as nebulizer gas (30 psi) and as turbo gas used for solvent drying (300 °C, 40 psi), whereas the curtain (20 psi) and collision gas (high) were nitrogen. Both quadrupoles were set at unit resolution, and the ion spray voltage was set at 5500 V in the positive mode. The different flavan-3-ols and the internal standard (chlorogenic acid) were detected and quantified from the signal of the corresponding transitions (each precursor ion–product ion pair) detected by multiple reaction monitoring analysis (MRM mode).^30^

### Salivary Protein Analysis by HPLC-DAD

Whole saliva (WS) was analyzed in the supernatant after the interaction assays by HPLC-DAD. All the samples, including the control solutions of each condition, were centrifuged prior to the chromatographic analysis. HPLC-DAD analysis was performed in an Agilent 1200 series HPLC system (Agilent Technologies, Palo Alto, CA) using a method previously optimized in our laboratory.^10^ Briefly, the stationary phase employed was a Zorbax 300SB-C8 5 μm column (2.1 × 150 mm), and the mobile phase was composed of solvent A (aqueous TFA 0.1%) and solvent B (TFA 0.1% in acetonitrile). The following gradient at a flow rate of 0.3 mL min^–1^ was used: 8–12% B in 10 min, 12–32% B in 50 min, followed by washing and re-equilibration of the column to initial conditions. The injection volume was 90 μL, and detection was carried out at 214 nm as the preferred wavelength.

### Statistical Analysis

To determine statistical significance of the differences between the absorbance values obtained in the DMACA assays, as well as the differences between the results from the quantification analysis, data were evaluated by one-way and two-way analysis of variance (ANOVA) and a posthoc Tukey-B test. In both cases, data were evaluated using the software packing for Windows IBM SPSS 26 (SPSS, Inc., Chicago, IL), where differences were considered statistically significant when *p* < 0.05.

## Results and Discussion

In recent years, the importance of developing experimental methods adapted to study the possible contribution of several mechanisms in the phenomenon of astringency has been revealed, and specifically the role of oral epithelial cells has gained prominence when it comes to mimicking the physiological situation. Furthermore, as the use of mannoproteins to modulate unbalanced astringency of red wines is of growing interest in the wine industry, in this work, we intended to develop an in vitro model of the oral cavity that allowed, for the first time, to simulate what happens in the mouth when drinking red wine that has been previously treated with mannoproteins. In this work, a purified extract of grape seed tannins was used as model tannins to simulate wine tannins. To achieve this objective, we have evaluated, in first place, the amount of flavan-3-ols that remain bonded to the oral cells after the interaction assays. Also, we have determined the amount of flavan-3-ols present in the supernatant after the interaction assays in each system and how the salivary profile is affected in the different systems studied.

In our study, four different systems were assayed: (1) oral cells + PBS (C+PBS), (2) oral cells + GSE (C+GSE), (3) oral cells + mannoprotein F or L (C+MPF or C+MPL), and (4) oral cells + GSE + mannoprotein F or L (C+GSE+MPF or C+GSE+MPL), in the presence or absence of saliva (S), as well as their respective controls without oral cells: (1) PBS, (2) GSE, (3) MPF or MPL, and (4) GSE + MPF or GSE + MPL, in the presence or absence of saliva (S).

### Interactions between Oral Cells and Tannins from GSE

**Figure 1:**
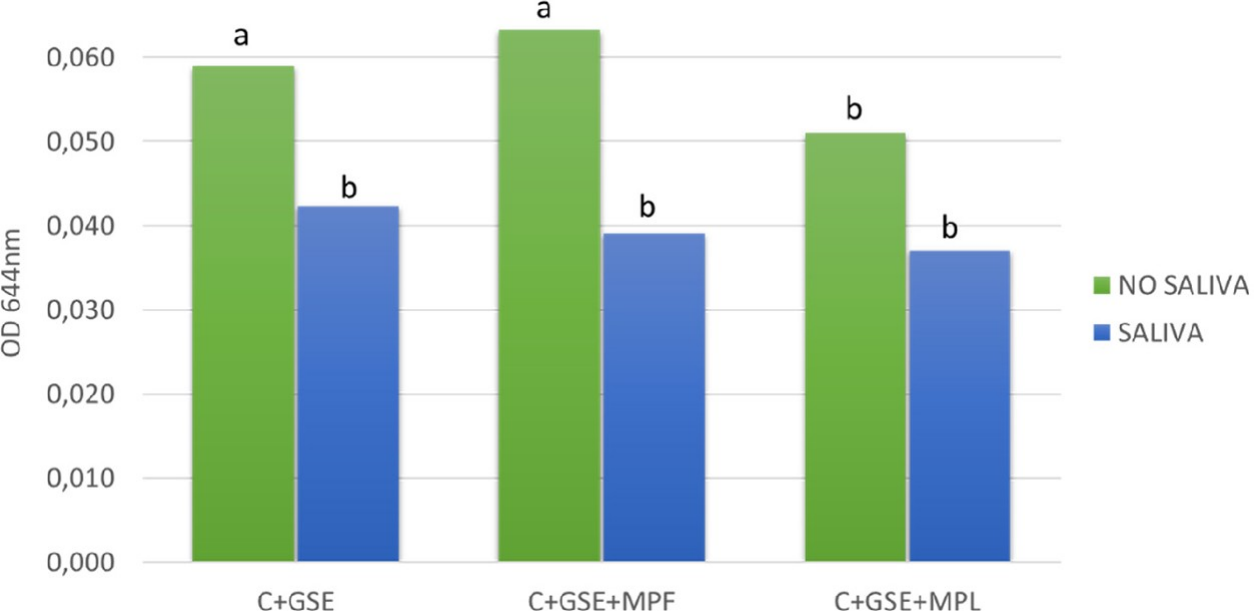
Absorbance values obtained in the DMACA assays evaluating the effect of saliva on the different systems: oral cells with flavan-3-ols from GSE (C+GSE), oral cells with flavan-3-ols and mannoprotein F (C+GSE+MPF), oral cells with flavan-3-ols and mannoprotein L (C+GSE+MPL). Different letters indicate statistical differences (*p* < 0.05) among samples.

After incubation of the cells with GSE in each condition, the supernatant of the cells was removed and the cells were washed with buffer, so just flavan-3-ols bound to the cells were determined by using DMACA assay. The absorbance values obtained after this procedure are presented in Figure 1. Significant differences between the absorbance values obtained in the different systems studied (C+GSE, C+GSE+MPF, and C+GSE+MPL), compared with the control wells of each system without cells, were found (data not shown). These results showed higher absorbance values obtained in the interaction assays when oral cells are present in the plate, thus confirming the existence of tannin–cell interactions, as previously reported elsewhere.^17,19^

Looking at the effect of the presence of saliva on cell–tannin interaction (Figure 1), we can see, in all the systems studied, a remarkable decrease in the absorbance obtained in the DMACA assay when saliva was added to the cell monolayer. This decrease was statistically significant in the systems C+GSE and C+GSE+MPF, pointing to a reduction of cell–tannin interactions caused by the presence of salivary proteins. This reduction of cell–tannin interactions was previously described by Ramos-Pineda and co-workers,^19^ who postulated that SP–tannin interactions predominate over tannin–cell binding. The highest decrease in the absorbance produced by the presence of saliva was obtained in the system containing MPF (C+GSE+MPF), which showed an inhibition of 38.2% compared with the system without saliva. These results could suggest a stronger interaction of salivary proteins with flavan-3-ols when this mannoprotein was present, therefore supporting the formation of protein/tannin/mannoprotein ternary soluble aggregates previously described for MPF.^27,36^ Also, these results pointed out that the protein/tannin/mannoprotein ternary soluble aggregates would not interact with the cells.

On the other hand, if we analyze the effect of the presence of the mannoproteins in the absence of saliva, although nonsignificant differences were found with MPF, the system C+GSE+MPF showed slightly higher values in the absorbance when compared with the system C+GSE (Figure 1), while the opposite occurred with MPL, showing significantly lower absorbance values related to flavan-3-ol binding to the cells. These results could suggest a different behavior of the two mannoproteins studied in their interaction with tannins, where MPL seems to reduce cell–tannin interactions. This MP could bind flavan-3-ols in the first place and prevent the subsequent cell–tannin interaction, exhibiting a behavior similar to that of salivary proteins. This would also be the reason why saliva addition to cell monolayers in the system C+GSE+MPL has no significant effect (Figure 1), because the presence of MPL would mask the effect of salivary proteins. However, further data would be needed to get specific details about this behavior.

The results obtained in the two-way analysis of variance (ANOVA) for the DMACA assay results are shown in Table 1. It can be observed that the presence of salivary proteins in the systems showed a remarkable effect on these interactions (*p* < 0.0001). Moreover, in the presence of saliva, the two MP studied seemed to have slight impact on cell–tannin interactions, as there was not a significant saliva–MP variation in any of the systems studied. However, in absence of saliva, MPL showed a significant effect (*p* = 0.006) by reducing cell–tannin interactions, while MPF seemed to have no effect, as the difference was not significant.

**Table 1:** Two-Way Analysis of Variance (ANOVA) Results for the Absorbance Values Obtained in the DMACA Assays: Evaluating the Effect of the Saliva (S) and the Mannoproteins F (MPF) and L (MPL) on the Different Systems Shown in Figure 1

source of variation	% of total variation	*P* value
S*	46.17	<0.0001
MPF	2.219	0.2152
MPL*	20.97	0.0060
S+MPF	1.666	0.2805
S+MPL	4.306	0.1789

After confirmation of the ability of the oral cells to interact with flavan-3-ols from the GSE and the influence of the presence of salivary proteins and mannoproteins on these interactions, further analyses were performed by HPLC-DAD to enhance the understanding of the mechanism whereby these biomolecules could modulate the interactions leading to the development of astringency.

### Salivary Protein Analysis by HPLC-DAD

The chromatographic conditions for the analysis of SP were previously optimized in our laboratory. Seven fractions were clearly separated and isolated, and their proteins and peptides were identified after tryptic digestion.^10^ To determine how the salivary protein profile is affected in the four systems studied (C+S+PBS, C+S+GSE, C+S+MPF+GSE, C+S+MPL+GSE), changes in the chromatographic areas of the different fractions were registered at 214 nm and were evaluated and compared to the control of whole mouth saliva (WS).

**Figure 2:**
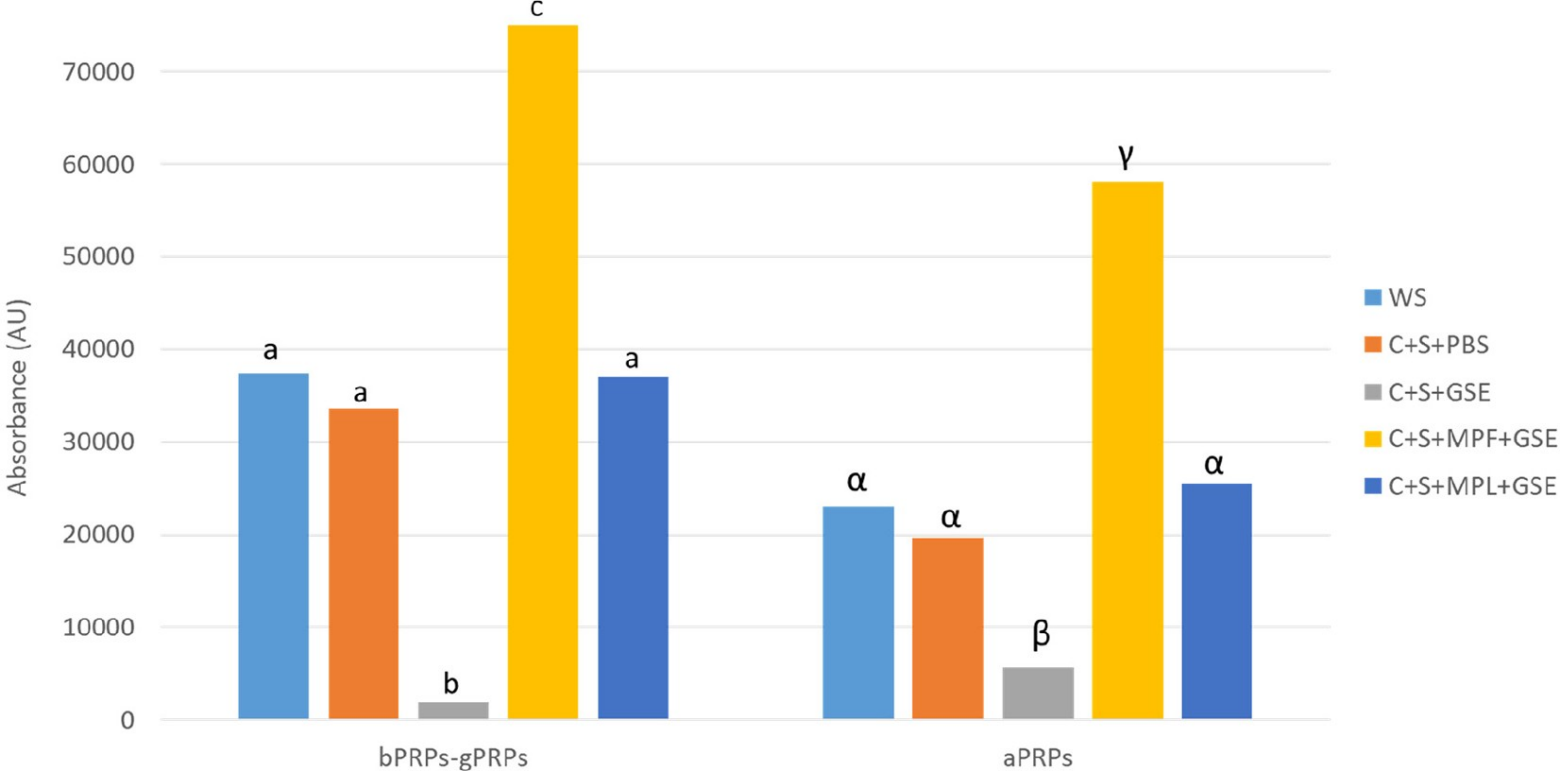
Chromatographic areas of salivary protein fractions (bPRPs-gPRPs and aPRPs) registered at 214 nm determined in the supernatant after the interaction assays. For each fraction, different letters indicate statistical differences (*p* < 0.05) among samples.

As proline-rich proteins (PRPs) seem to be the most important component of saliva with regards to astringency,^6^ in this work we have analyzed changes in the fraction mainly composed of basic PRPs, both nonglycoslylated (bPRPs) and glycosylated (gPRPs), and in the fraction corresponding to acidic PRPs (aPRPs). The chromatographic areas obtained are presented in Figure 2, showing large variations between the different systems studied. The system C+S+GSE shows a very significant decrease in the chromatographic areas, achieving values of 94.8% decrease in the area corresponding to the fraction of bPRPs-gPRPs and 75.5% decrease for aPRPs. These values seem to support the precipitation of protein–tannin aggregates after the incubation of salivary proteins with flavan-3-ols from GSE and subsequent centrifugation, leading to a reduction of the area corresponding to these proteins. Focusing on the role of the mannoproteins, it is noteworthy that the highest values in the chromatographic areas were obtained with MPF in the two protein fractions (system C+S+MPF+GSE), being even more pronounced for the fraction of bPRPs-gPRPs, possibly because of the formation of soluble complexes with this mannoprotein. This result is in good agreement with the formation of large ternary soluble complexes between the salivary proteins, mannoprotein, and flavan-3-ols, previously proposed for MPF.^27,36^ However, looking at the areas in the system C+S+MPL+GSE, very similar areas were obtained when compared to the control WS. Thus, it seems that MPL could prevent the interaction between salivary proteins and flavan-3-ols by a competitive mechanism, i.e., flavan-3-ols could interact with both MPL and salivary proteins, which might increase the number of total binding sites available for flavan-3-ols. As a result, the number of flavan-3-ols bound per salivary protein decreases, which would affect the aggregation, because aggregation requires several flavan-3-ols per protein to occur.^37^

### Flavan-3-ol Quantification by HPLC-MS

**Figure 3:**
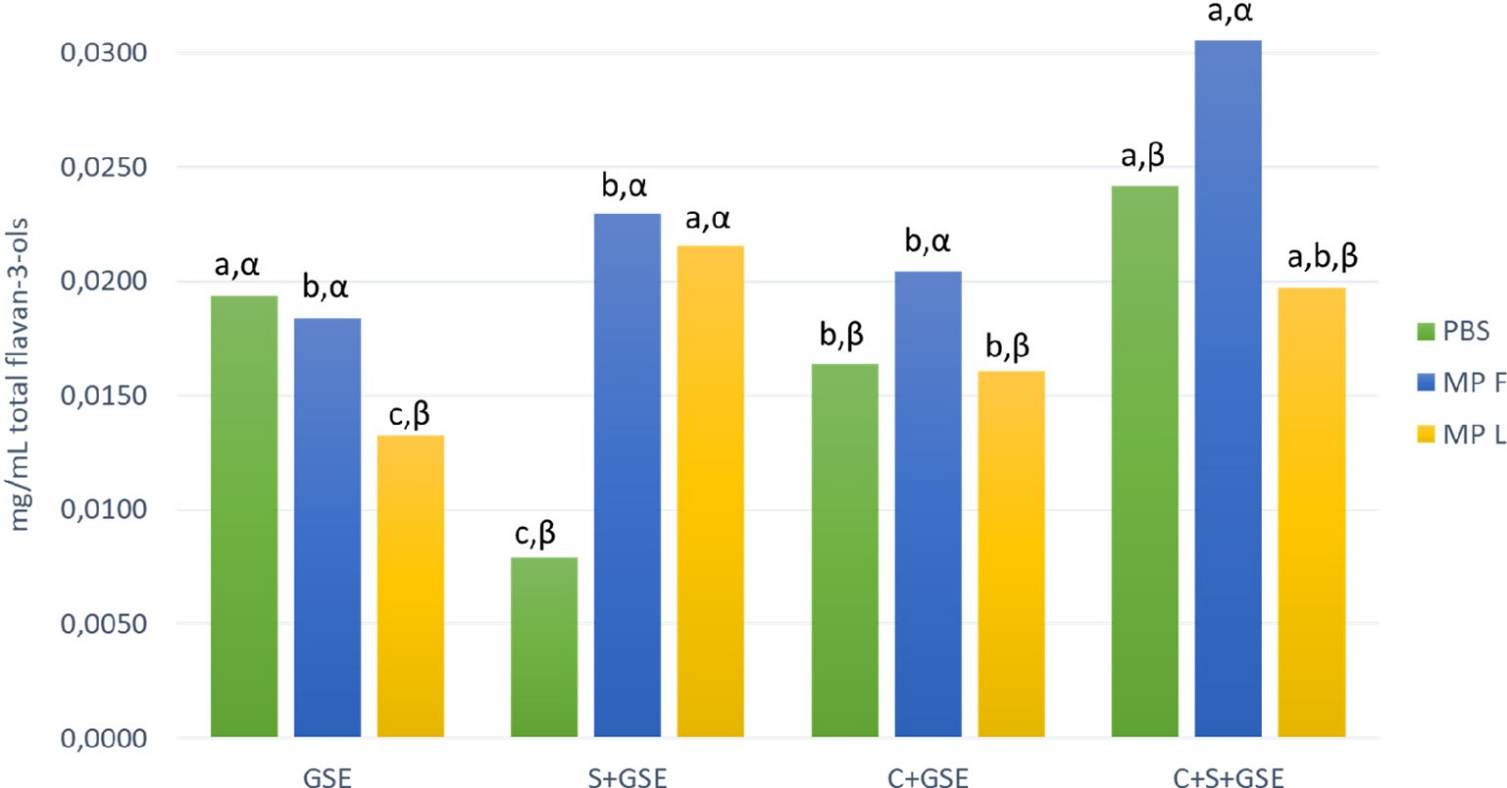
Total content of flavan-3-ols (mg/mL) determined in the supernatant after the interaction assays, obtained by HPLC-DAD-MS quantification, evaluating the effect of the mannoproteins F (MPF) and L (MPL) on the different systems: control condition without oral cells with flavan-3-ols (GSE), salivary proteins with flavan-3-ols (S+GSE), oral cells with flavan-3-ols (C+GSE), and oral cells with salivary proteins and flavan-3-ols (C+S+GSE). Different Roman letters indicate statistical differences (*p* < 0.05) among samples in the four systems (GSE, S+GSE, C+GSE, and C+S+GSE) while different Greek letters indicate statistical differences (*p* < 0.05) among samples in the different treatments (PBS, MPF, and MPL).

After the interaction assays, the supernatant from the wells was collected, centrifuged, and then analyzed by HPLC-DAD-MS. Figure 3 shows the concentration of total flavan-3-ols obtained in the supernatant after the interaction assays performed in the different systems (GSE, S+GSE, C+GSE, and C+S+GSE), comparing the concentration obtained in each system in the presence or absence of mannoprotein F or L. As for the system only with GSE (without cells), first, it should be stressed that the total flavan-3-ols value obtained in GSE+PBS does not necessarily represent the overall amount of flavan-3-ols present in the extract. Self-aggregation and precipitation of flavan-3-ols has been previously reported by different authors,^38−40^ which may cause the precipitation of tannin–tannin aggregates during centrifugation, resulting in a lower concentration value than the actual flavan-3-ol composition in the GSE used. The addition of MPL showed a lower statistically significant value in the flavan-3-ol concentration compared with GSE+PBS, suggesting again that this mannoprotein favors the formation of insoluble aggregates. This explanation appears to be consistent with other studies, where the interaction between tannins from a flavan-3-ol extract and MPL was analyzed by isothermal titration calorimetry (ITC) and molecular dynamics (MD).^27^ Thus, these tannin–MP aggregates could explain the lower value in the flavan-3-ol concentration observed in the supernatant.

If we focus on the system S+GSE (without cells), we can observe lower values in the flavan-3-ol concentration obtained with PBS. The formation of soluble and insoluble aggregates between salivary proteins and flavan-3-ols has been extensively described by many authors,^10,41,42^ explaining the low concentration values observed in the system S+GSE with PBS. However, statistically significant differences can be observed in the flavan-3-ols content due to the presence of both MPF and MPL, although MPF seemed to have a slightly stronger effect. These higher values seem to confirm the formation of SP/MPF/flavan-3-ol ternary soluble aggregates, considered as one of the main mechanisms to modulate astringency by the use of polysaccharides.^27,43^ Moreover, as previously explained, MPL could prevent the flavan-3-ol–saliva interaction by a competitive mechanism, because this MP, due to their longer peptide chains, may have more binding sites and could thus be more effective to bind tannins, which could also explain the differences in the concentration values obtained.

On the other hand, some interesting differences can be highlighted when we compare the system C+GSE with the system GSE in the absence of oral cells. Lower flavan-3-ol concentration values were observed when oral cells were present, which could be expected due to cells–tannin interactions previously seen in the DMACA assays. These data confirm the existence of cell–tannin interactions, although these interactions seem to be less important than salivary protein–tannin interactions. Moreover, some differences can be highlighted when we analyze the behavior of these mannoproteins in the system C+GSE. A different trend in the flavan-3-ol concentration produced by the two MP assayed can be noted. In this case, the presence of MPF led to higher values in the total flavan-3-ols, as expected because MPF seems to act by favoring the solubility of flavan-3-ols, possibly through the formation of soluble aggregates, which would protect flavan-3-ols to interact with oral cells. Moreover, the formation of these ternary soluble aggregates in the presence of MPF seems to be more important in the system C+S+GSE than in the system S+GSE, pointing out a possible interaction between salivary proteins and oral cells that modify the interaction between SP, MPF, and flavan-3-ols in the solution. By contrast, MPL appeared to have no effect compared with the system C+GSE+PBS. However, on the basis of the results discussed above, it could be hypothesized that, in this system, a possible MPL–flavan-3-ol interaction could be taking place, reducing cell–tannin interactions and leading to the precipitation of MP–tannin aggregates. Again, the behavior of this mannoprotein could be compared to that of salivary proteins in the inhibition of cell–tannin interactions, although MPL would have much lower precipitation capacity.

Looking at the system C+S+GSE, we observed, again, the reduction of cell–tannin interactions produced by the presence of saliva, leading to higher values of flavan-3-ol content. Regarding this system with MP, we can highlight the effect of MPF, showing the highest flavan-3-ol concentration value compared with all the systems studied. The formation of ternary soluble aggregates between salivary proteins, mannoproteins, and flavan-3-ols has been previously demonstrated,^36^ avoiding the precipitation of these aggregates during the centrifugation step, which might explain this higher value. For its part, the system C+S+GSE with MPL showed lower concentration values than with MPF and very similar to that obtained for GSE+PBS.

**Figure 4:**
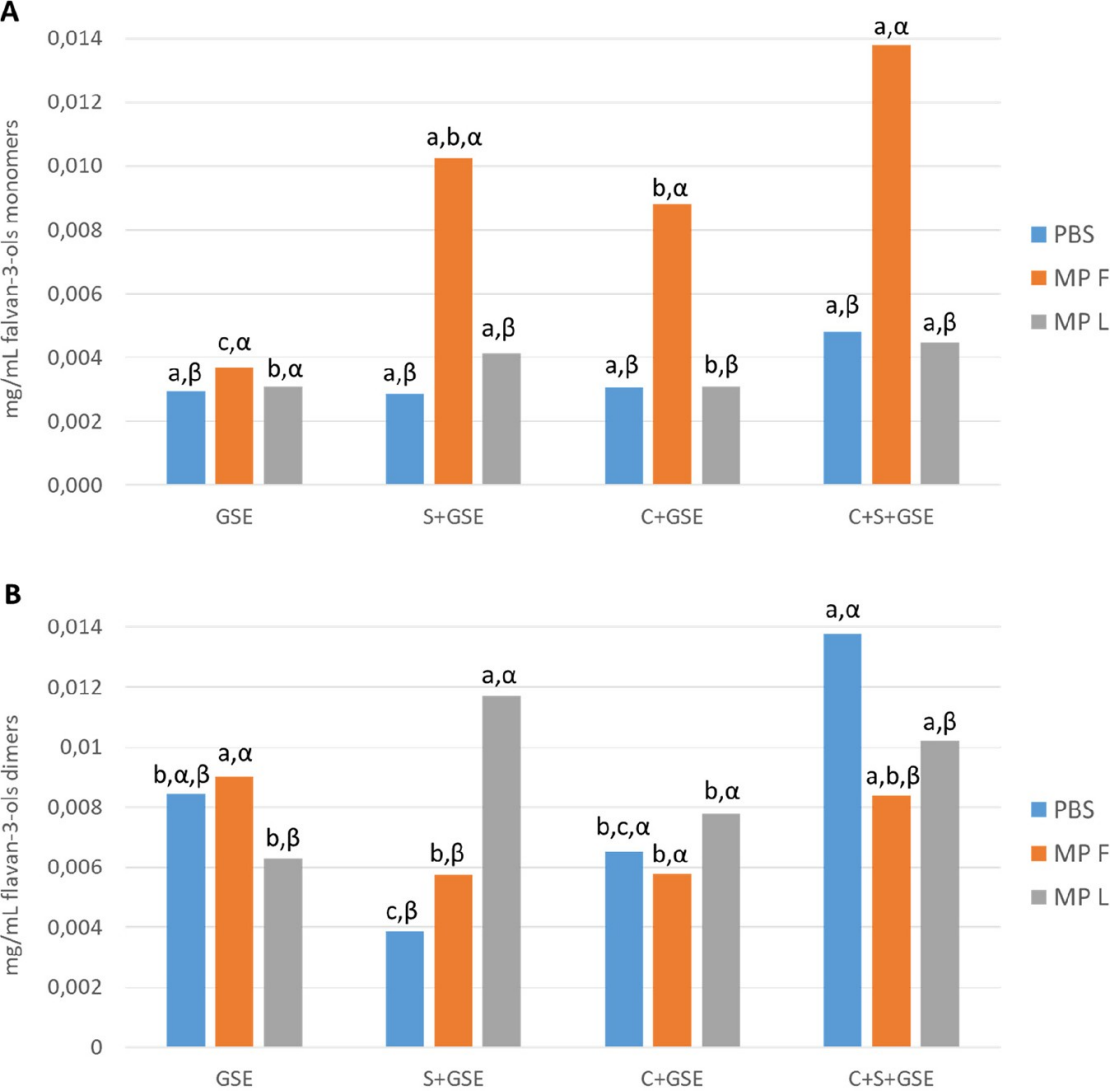
Content of flavan-3-ol monomers (A) and dimers (B) (mg/mL) obtained by HPLC-DAD-MS quantification after the interaction assays, evaluating the effect of the mannoproteins F and L on the different systems: control condition without cells, oral cells with flavan-3-ols (GSE), salivary proteins with flavan-3-ols (S+GSE), oral cells with flavan-3-ols (C+GSE), and oral cells with salivary proteins and flavan-3-ols (C+S+GSE). Different Roman letters indicate statistical differences (*p* < 0.05) among samples in the four systems (GSE, S+GSE, C+GSE, and C+S+GSE) while different Greek letters indicate statistical differences (*p* < 0.05) among samples in the different treatments (PBS, MPF, and MPL).

After analyzing the differences previously seen in the concentration of total flavan-3-ols present in the supernatant after interaction assays, we also studied the behavior of the two main families found in the GSE (Figure 4), monomeric and dimeric flavan-3-ols (the sum of monomers and dimers represent more than 70% of total flavan-3-ols in GSE, see Table SI-1 of the Supporting Information), to enhance the knowledge of the behavior of the different flavan-3-ols in the studied systems and even if the two MP studied could have different affinities for each type of flavan-3-ol.

There were some differences between the behavior of the monomers and dimers that can be highlighted. First, it could be observed that interactions of dimeric flavan-3-ols with salivary proteins are slightly different in the presence than in the absence of oral cells, pointing out that some salivary proteins could interact with oral cells,^44^ which may modify the behavior of salivary proteins toward this type of flavan-3-ol. Moreover, although the presence of MPF could favor the solubility of both monomers and dimers in the supernatant after the interaction with SP, the formation of the ternary soluble aggregates that would explain this fact seems to be more important for monomers than for dimers in the systems S+GSE, C+GSE, and C+S+GSE. As for MPL, its behavior with monomers is similar to that observed for total flavan-3-ols, but also some differences were found when we looked into dimers. In this case, the competitive mechanism previously observed could also take place, because MPL leads to the precipitation of dimeric flavan-3-ols, suggesting again the formation of insoluble MPL–tannin binary aggregates (GSE+MPL system). However, it seems that ternary soluble aggregates involving MPL, salivary proteins, and dimeric flavan-3-ols could also be formed.

To conclude, the results presented herein showed a different behavior between the two mannoproteins studied in the presence of oral cells, which suggests different mechanisms of action. The low protein percentage mannoprotein (MPF) seems to promote flavan-3-ol solubility, probably due to the formation of ternary soluble aggregates, and protects them from interaction with oral cells.

By comparison, MPL, which is a mannoprotein with high protein content, has shown to behave very differently than MPF. As previously mentioned, MPL has lower affinity for salivary proteins and stands out for its interaction with flavan-3-ols, giving rise to the formation of mannoprotein–flavan-3-ol binary complexes. Thus, the main mechanism suggested to explain its modulating effect on astringency has been a competitive mechanism.^27^ On the basis of the results obtained in this work, it seems that MPL forms MP–tannin binary aggregates. Furthermore, MPL has shown a very particular behavior; in the systems with oral cells it has demonstrated reduced cell–tannin interactions, and this inhibitory behavior could be compared (to a lesser extent) to that of salivary proteins. Moreover, MPL also prevents saliva–tannin interactions as suggested by the competitive mechanism previously proposed. Then, in the context of the oral cavity, this mannoprotein could reduce the astringency caused by tannins through different mechanisms that could be taking place at the same time. To our knowledge, this is the first time that a reduction in the interaction between grape procyanidins and oral cells by a mannoprotein has been revealed.

Both mannoproteins have shown their ability to affect salivary protein–flavan-3-ol interactions, preventing the precipitation of saliva–tannin complexes (by the formation of ternary complexes or by a competitive mechanism). Saliva–tannin interactions appear to be much more important than cell–tannin interactions, and it seems clear that the presence of saliva prevents cell–tannin interactions.

Moreover, it has been observed that mannoproteins with different compositional characteristics could exhibit a preferential interaction with some flavan-3-ol families. In this case, the low protein percentage (MPF) seems to protect monomers from their precipitation, while the high protein content (MPL) has shown a greater tendency to interact with dimers of flavan-3-ols.

This is the first time that the role of mannoproteins in the interaction between salivary proteins and flavan-3-ols has been studied in the context of an in vitro model of the oral cavity. Most of the studies published to date focused on the study of the mechanisms whereby mannoproteins reduce wine astringency and only considered the presence of salivary proteins and procyanidins in the system; however, here the importance of including oral cell models when studying this phenomenon has been revealed. Finally, we highlight the importance of considering more than one mechanism when studying not only the origin of astringency but also the modulating effect of mannoproteins.

## Supporting Information Available

Composition of flavan-3-ols in grape seed extract. Schematic representation of a 96-well plate of the interaction assays (PDF)
