# Supplementary material for: Role of Yeast Mannoproteins in the Interaction between Salivary Proteins and Flavan-3-ols in a Cell-Based Model of the Oral Epithelium
Source: J Agric Food Chem. 2022 May 31;70(41):13027–35. doi: 10.1021/acs.jafc.1c08339 (PMC9585572; doi:10.1021/acs.jafc.1c08339)

Supporting Information

Table SI-1. Composition (%) of flavan-3-ols in grape seed extract determined by HPLC-DAD-MS.

	**Non galloylated**	**Galloyl derivatives**
**Monomers**	23.58	1.43
**Dimers**	43.01	3.86
**Trimers**	17.89	1.85
**Tetramers**	6.73	0.97
**Pentamers**	0.61	0.06
**TOTAL**	91.82	8.17

Figure S1. Scheme representation in a 96-well plate of the interaction assays between flavan-3-ols (GSE), mannoproteins (MP) and oral epithelial cells in presence/absence of salivary proteins. Columns 1−6 correspond to the wells with PBS (no saliva) while columns 7-12 correspond to the wells where saliva was present. Lines A and H represent the control conditions without oral cells.

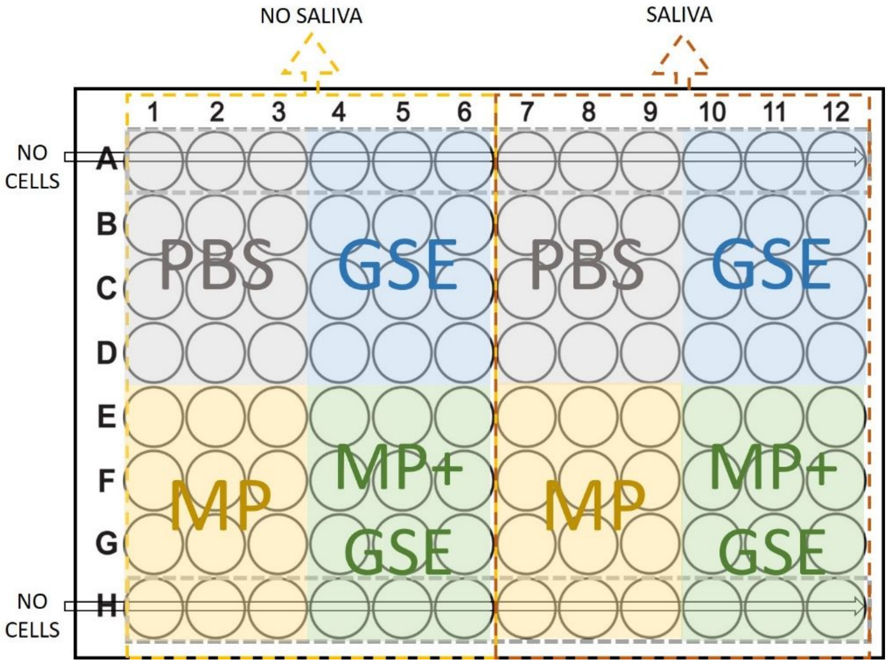

Supplement: Supplementary file 1 — jf1c08339_si_001.pdf [file jf1c08339_si_001.pdf]
